# Protective Role of Ashwagandha Leaf Extract and Its Component Withanone on Scopolamine-Induced Changes in the Brain and Brain-Derived Cells

**DOI:** 10.1371/journal.pone.0027265

**Published:** 2011-11-11

**Authors:** Arpita Konar, Navjot Shah, Rumani Singh, Nishant Saxena, Sunil C. Kaul, Renu Wadhwa, Mahendra K. Thakur

**Affiliations:** 1 Biochemistry and Molecular Biology Laboratory, Department of Zoology, Banaras Hindu University, Varanasi, India; 2 National Institute of Advanced Industrial Science and Technology (AIST), Tsukuba, Ibaraki, Japan; Alexander Flemming Biomedical Sciences Research Center, Greece

## Abstract

**Background:**

Scopolamine is a well-known cholinergic antagonist that causes amnesia in human and animal models. Scopolamine-induced amnesia in rodent models has been widely used to understand the molecular, biochemical, behavioral changes, and to delineate therapeutic targets of memory impairment. Although this has been linked to the decrease in central cholinergic neuronal activity following the blockade of muscarinic receptors, the underlying molecular and cellular mechanism(s) particularly the effect on neuroplasticity remains elusive. In the present study, we have investigated (i) the effects of scopolamine on the molecules involved in neuronal and glial plasticity both *in vivo* and *in vitro* and (ii) their recovery by alcoholic extract of Ashwagandha leaves (i-Extract).

**Methodology/Principal Findings:**

As a drug model, scopolamine hydrobromide was administered intraperitoneally to mice and its effect on the brain function was determined by molecular analyses. The results showed that the scopolamine caused downregulation of the expression of BDNF and GFAP in dose and time dependent manner, and these effects were markedly attenuated in response to i-Extract treatment. Similar to our observations in animal model system, we found that the scopolamine induced cytotoxicity in IMR32 neuronal and C6 glioma cells. It was associated with downregulation of neuronal cell markers NF-H, MAP2, PSD-95, GAP-43 and glial cell marker GFAP and with upregulation of DNA damage- γH2AX and oxidative stress- ROS markers. Furthermore, these molecules showed recovery when cells were treated with i-Extract or its purified component, withanone.

**Conclusion:**

Our study suggested that besides cholinergic blockade, scopolamine-induced memory loss may be associated with oxidative stress and Ashwagandha i-Extract, and withanone may serve as potential preventive and therapeutic agents for neurodegenerative disorders and hence warrant further molecular analyses.

## Introduction

Memory function is vulnerable to a variety of pathological processes including neurodegenerative diseases, stroke, tumors, head trauma, hypoxia, cardiac surgery, malnutrition, attention-deficit disorder, depression, anxiety, side effects of medication, and aging [1]. Severe memory loss and deficits in broad range of cognitive abilities are commonly categorized as age-pathology because of its frequent occurrence in later part of human lifespan. Memory loss is often the most disabling feature of many disorders, impairing the normal daily activities of the patients and profoundly affecting their families. Animal models of memory impairment have been used to understand its molecular basis and search its therapeutic targets. Scopolamine- induced amnesia rodent model is one of the well-established animal models of memory dysfunction [2]. Scopolamine is a non-selective muscarinic receptor antagonist that inhibits central cholinergic neuronal activity and impairs learning and short-term memory. It influences the expression of a broad spectrum of genes including those associated with muscarinic receptor signaling pathways, apoptosis, cytoskeleton reconstruction, protein trafficking and cell differentiation in rat brain [3]. However, molecular details and the role of these signaling pathways remain undefined [4].

Memory is tightly associated with brain plasticity related events at different levels and involves changes in the expression of molecules, intracellular signaling cascades, synaptic strength and neural networks. Brain plasticity is regulated by several factors including brain derived neurotrophic factor (BDNF), a member of the neurotrophin family of growth factors that is widely expressed throughout the mammalian brain along with its high affinity receptor TrkB [5] and plays a crucial role in development, maintenance and function of the central nervous system (CNS). Accumulating data have suggested that the neuronal activity regulates BDNF transcription, transport to dendrites and secretion that in turn modulates synaptogenesis, synaptic plasticity and memory formation [6]. Furthermore, it has been established that glial cells and their resident protein glial fibrillary acidic protein (GFAP) integrate neuronal input, modulate synaptic activity and process signals related to learning and memory by formation of cytoskeletal filaments [7]. It provides structural support to neurons and is necessary for CNS morphogenesis [8] and hence is considered as a marker of glial plasticity controlling the structure, adhesion and proliferation of astrocytes, neuron-glia interactions and mechanisms of memory formation. Functional impairment and modifications of these molecules that signal and articulate memory function are widely considered in development of nootropics including drugs, supplements, biomolecules and their derivatives. One of the widely used herbal extract includes Ashwagandha (*Withania somnifera*) that has been shown to enhance neuritic regeneration and synaptic reconstruction. However, the molecular mechanism of such activity is not well understood. In the present study, we have investigated the neuroprotective potential of the alcoholic extract of Ashwagandha leaves (i-Extract) that is rich in withaferin A and withanone (i-Factor) in scopolamine-induced amnesia mouse and brain cell culture (IMR32, neuronal and C6, glioma) models.

## Results

### Scopolamine affected the BDNF and GFAP mRNA expression in mouse cerebrum in a dose and time dependent manner

**Figure 1:**
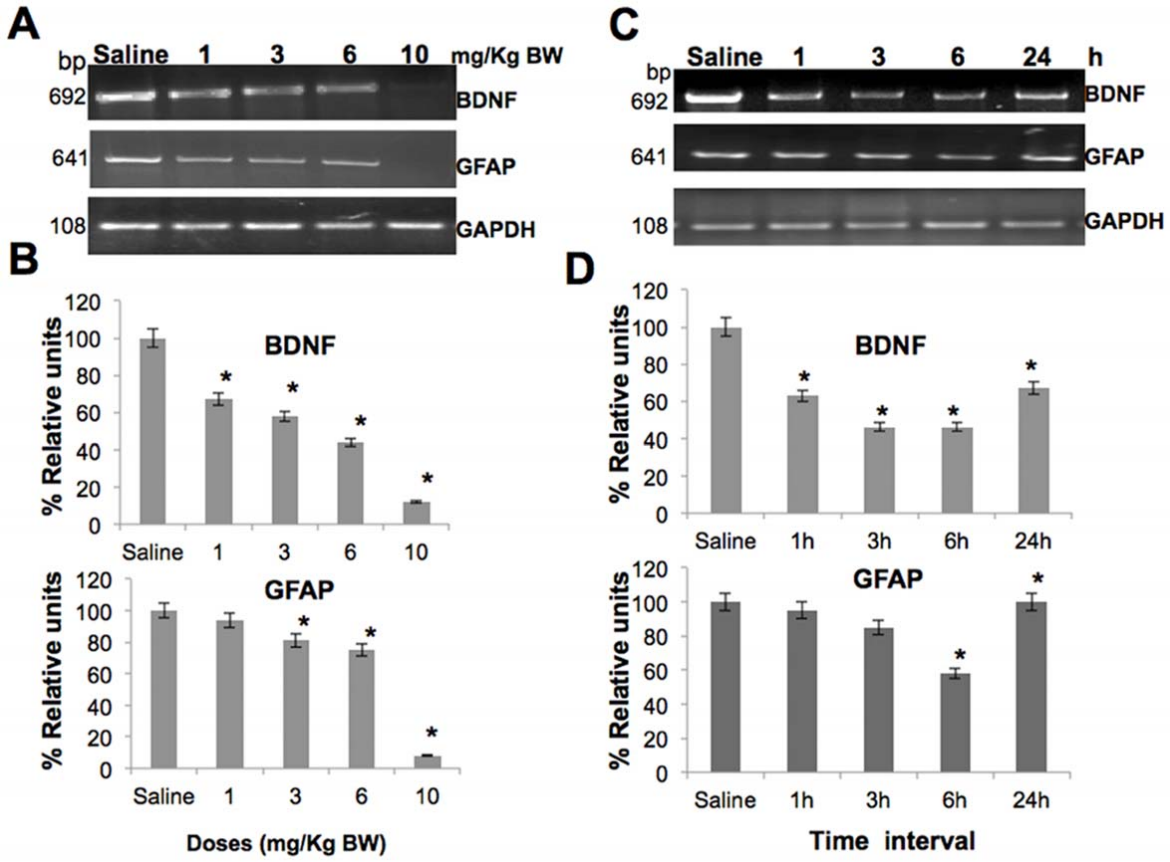
Effect of scopolamine on BDNF and GFAP expression in mice brain. Dose and time dependent effects of scopolamine (SC- 1, 3, 6, 10 mg/Kg BW) on the level of expression of BDNF and GFAP mRNA in mouse cerebrum: (A) RT-PCR analysis showing BDNF (692 bp), GFAP mRNA (641 bp) and internal control GAPDH (108 bp). The band intensity was quantitated densitometrically (represented as relative units) by normalizing with GAPDH) from three independent experiments (±) SEM. (B) Histogram represents percent relative units of BDNF and GFAP expressions from three independent experiments (±) SEM. *denotes significant differences (P<0.05) between the groups. (C) Time (1, 3, 6, 24 h) dependent effect of scopolamine (SC- 3 mg/Kg BW) on expression of BDNF and GFAP mRNA in mouse cerebrum. Data was quantitated (D) as mentioned above.

In order to determine the effect of scopolamine on neuroplasticity, we analyzed BDNF (transcript variant-1) and GFAP mRNA in mouse cerebrum. In RT-PCR analysis, 692 nucleotides fragment of BDNF mRNA and 641 nucleotides fragment of GFAP mRNA were amplified. We found that both BDNF and GFAP were downregulated in scopolamine treated mouse cerebrum. Furthermore, the effect was dose dependent. As compared to control saline treatment (set at 100%), scopolamine treated mice showed dose dependent drastic reduction in expression level of BDNF (1 mg/kg BW-67%, 3 mg/kg BW-58%, 6 mg/kg BW- 44% and 10 mg/kg BW-12%; P<0.05) (Figure 1, A and B). Similarly, GFAP message was reduced in a dose dependent, but moderate manner (1 mg/kg BW-94%, 3 mg/kg BW-81%, 6 mg/kg BW- 75% and 10 mg/kg BW-8% P<0.05) (Figure 1A and 1B). The difference in BDNF was found to be statistically significant between control and treated groups. However, for GFAP message, all the groups, except 1 mg/kg BW group, showed significant alteration. Since BDNF and GFAP have been shown to be involved in regulation of neuronal plasticity and differentiation, these data suggested that the scopolamine affected neuroplasticity.

In order to investigate whether scopolamine mediated effect on neuroplasticity was long lasting or transient, the expression level of BDNF and GFAP was examined after 1, 3, 6 and 24 h of scopolamine treatment; 3 mg/kg BW that resulted in 40% decrease in BDNF in comparison to only 15–20% for GFAP and has been considered physiologically relevant in other studies [9]–[12] was selected. As seen in Figure 1C and 1D, down-regulation of BDNF and GFAP occurred in a time-dependent manner. As compared to the untreated saline control (set at 100%), BDNF expression level gradually decreased until 6 h and thereafter it showed increase at 24 h (1 h- 63%, 3 h- 50%, 6 h- 46% and 24 h- 67% P<0.05). Statistical analysis revealed that the change between control and treated groups, except between 3 h and 6 h, were statistically significant (Figure 1C and 1D). Similarly, GFAP message level also showed time-dependent reduction until 6 h and thereafter it increased to control level by 24 h (1 h- 95%, 3 h- 85%, 6 h- 58% and 24 h-100% P<0.05). In case of GFAP, comparison was statistically significant between control and treated groups except between the control and 1 h, and control and 24 h groups (Figure 1C and 1D). The data from this pilot study implied that the scopolamine-mediated effects on BDNF and GFAP expression were transient and lasted for nearly 24 h.

### Effect of i-Extract on scopolamine-induced downregulation of BDNF and GFAP in mouse cerebrum

**Figure 2:**
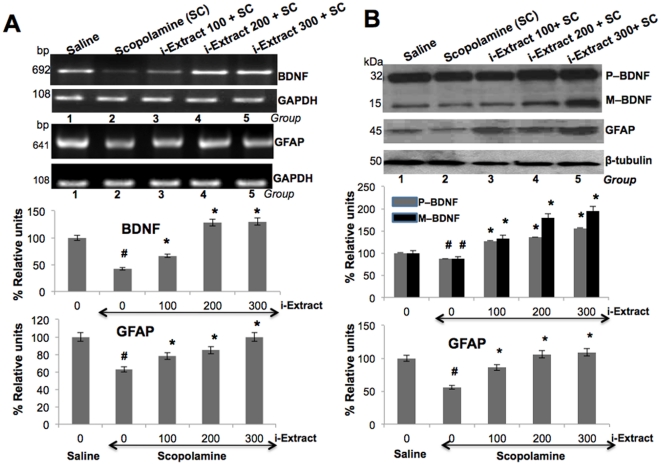
Ashwagandha i-Extract treatment and reversal of scopolamine-induced decrease in BDNF and GFAP expression in mice brain. Effects of i-Extract (100, 200, 300 mg/Kg BW) on scopolamine (SC- 3 mg/Kg BW) induced decrease in mRNA (A) and protein (B) expression levels of BDNF and GFAP in mouse cerebrum. Data was quantitated and is shown as percent relative units. *denotes significant differences (P<0.05) between the SC group and i-Extract group. #denotes significant differences (P<0.05) between the control and SC group.

To assess the effects of i-Extract on scopolamine-mediated decrease in levels of BDNF and GFAP, we analyzed both message and protein level of BDNF and GFAP in cerebrum samples from mice that were treated with i-Extract and scopolamine. Expression analysis of BDNF mRNA (Figure 2A) showed that the scopolamine reduced its level to about half; and pretreatment of i-Extract markedly attenuated the decrease by 50% (Group 3- i-Extract 100 mg/kg BW) and 200% in Group 4 (i-Extract 200 mg/kg BW) and Group 5 (i-Extract 300 mg/kg BW). In the western blot analysis, anti-BDNF polyclonal rabbit antiserum detected pro-BDNF as ∼32-kDa and mature BDNF as ∼15-kDa (Figure 2B) and revealed a pattern similar to BDNF mRNA; although the extent of alteration was milder. Scopolamine reduced both precursor and mature BDNF protein to about 3/4 level that was statistically significant and i-Extract attenuated the decrease by 27% for pro-BDNF and 33% for mature BDNF in Group 3 (i-Extract 100 mg/kg BW), 36% for pro BDNF and 80% for mature BDNF in Group 4 (i-Extract 200 mg/kg BW) and 56% for pro BDNF and 95% for mature BDNF in Group 5 (i-Extract 300 mg/kg BW) P<0.05.

**Figure 3:**
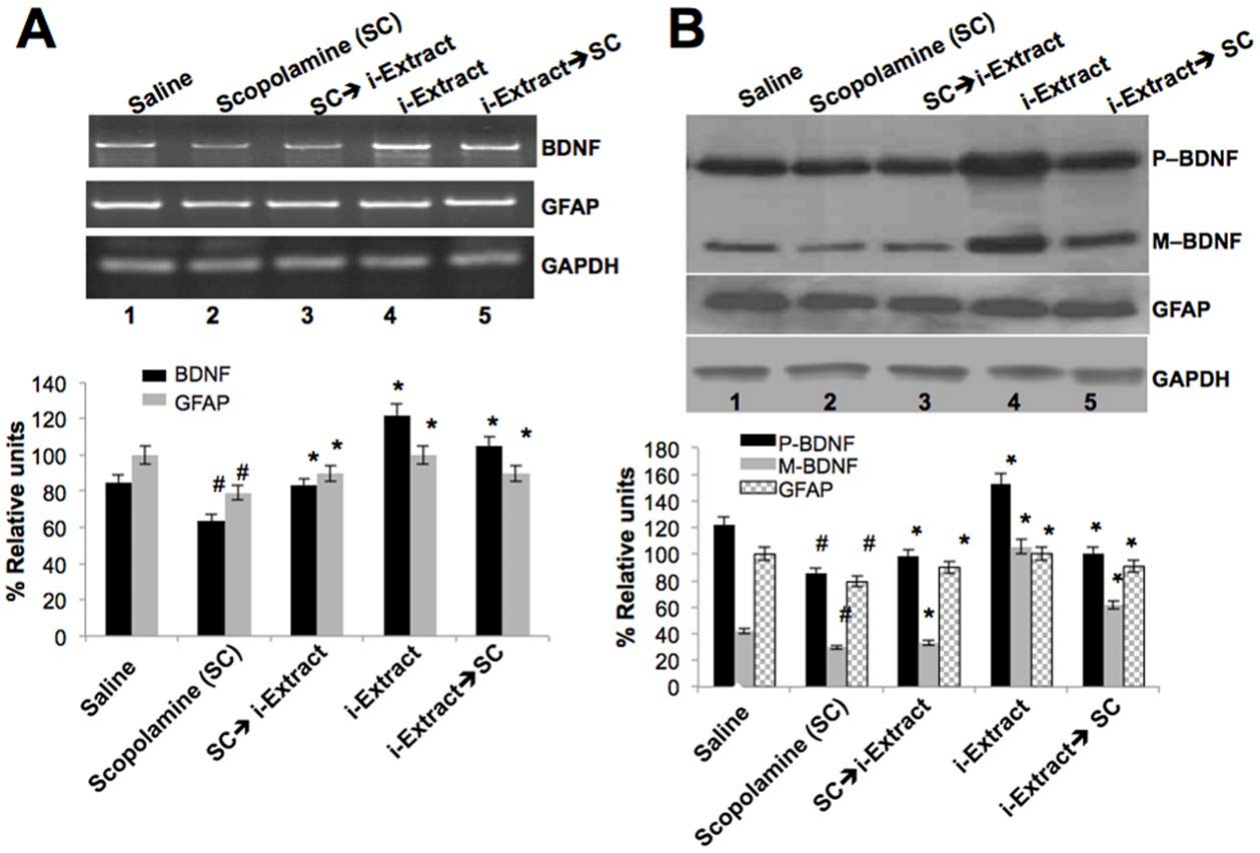
Effect of pre- and post-treatment with Ashwagandha i-Extract on scopolamine-induced changes in BDNF and GFAP expression in mice brain. Effects of pre, post and *per se* treatment of i-Extract (200 mg/Kg BW) on mRNA expression (A) and protein (B) levels of BDNF and GFAP in the mouse cerebrum. Data was quantitated and is presented as percent relative units. Lane 1-Control, Lane 2-scopolamine, Lane 3-post-treatment with i-Extract, Lane 4-*per se* treatment of i-Extract, Lane 5-Pre-treatment with i-Extract. *denotes significant differences (P<0.05) between the SC group and i-Extract group. #denotes significant differences (P<0.05) between Control and SC group.

Expression analysis of GFAP message (Figure 2A) showed that the scopolamine reduced its level to about 3/4th whereas i-Extract attenuated the decrease by 24% (Group 3, i-Extract 100 mg/kg BW), 35% (Group 4, i-Extract 200 mg/kg BW) and 58% (Group 5, i-Extract 300 mg/kg BW P<0.05). Effect of i-Extract on GFAP protein (Figure 2B) followed a similar pattern. In the Western blot analysis, anti-GFAP polyclonal rabbit antiserum recognized GFAP of ∼45-kDa. Scopolamine reduced GFAP protein level to about half and i-Extract attenuated the decrease by 54% (Group 3, i-Extract 100 mg/kg BW), 88% (Group 4, i-Extract 200 mg/kg BW) and 93% (Group 5, i-Extract 300 mg/kg BW) P<0.05. Moreover, the effect of pre- and post-treatment with i-Extract (200 mg/kg BW) on scopolamine (3 mg/kg BW)-induced alterations were also examined. In pre-treatment, mice were first treated with i-Extract followed by scopolamine, while in post-treatment mice were treated first with scopolamine followed by i-Extract. The data revealed that whereas the pretreatment of i-Extract reverted the scopolamine-induced decrease in BDNF, the post-treatment was ineffective (Figure 3A and 3B). GFAP message and protein level (Figure 3A and 3B) were increased by i-Extract treatments (both pre and post) (Figure 3A and 3B). Furthermore, we found that the administration of i-Extract, without scopolamine upregulated the expression of BDNF mRNA as well as the protein suggesting that the i-Extract has factors that may enhance neuroplasticity. The data revealed that pre-treatment of i-Extract was more effective in attenuating scopolamine-induced downregulation of BDNF message and protein as compared to post-treatment of i-Extract. Moreover, the administration of i-Extract alone, without scopolamine, upregulated BDNF message and protein level. In case of GFAP, both pre- and post- treatment of i-Extract equally attenuated scopolamine induced downregulation but to a relatively lower extent as compared to BDNF.

### Effect of i-Extract on scopolamine-induced toxicity to glial and neuronal cells

**Figure 4:**
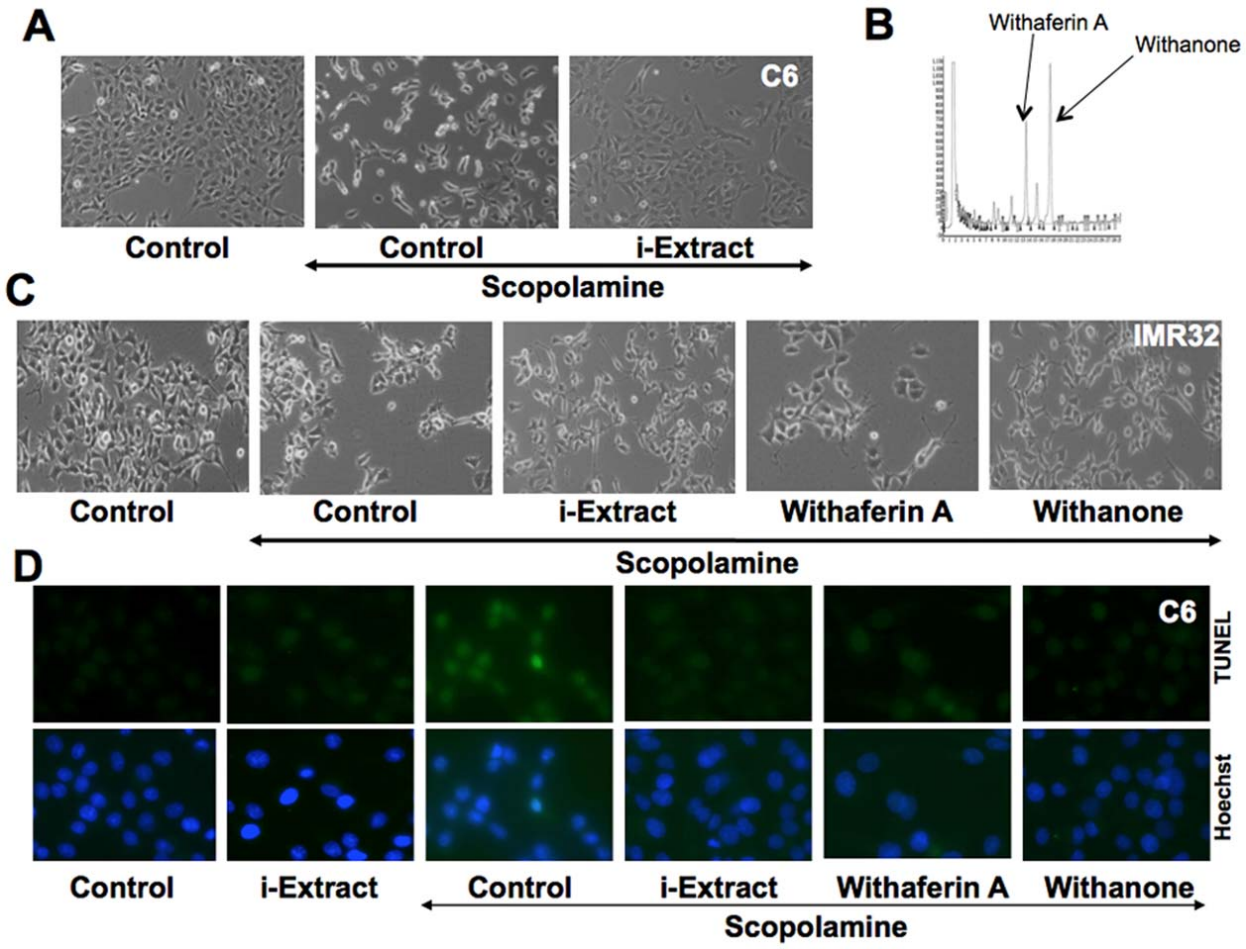
Effect of i-Extract on scopolamine-induced toxicity in C6 glioma and IMR32 neuroblastoma cells. (A) i-Extract treatment resulted in increased viability of C6 cells. (B) Composition of i-Extract (the content of Withaferin A and Withanone is shown. (C) IMR32 cells treated with scopolamine and i-Extract, Withaferin A and Withanone (i-Factor) supplemented medium are shown. (D) TUNEL assay of the control and treated cells (as indicated) showed apoptosis in scopolamine treated cells. Pre-treatment of cells and recovery in i-Extract supplemented medium showed downregulation of scopolamine induced apoptosis.

In order to understand the molecular mechanism of scopolamine-induced toxicity and its recovery in response to i-Extract treatment, and to define the active component for neuroprotection, we adopted glioma and neuronal cell culture models and examined their effects on cell survival. As shown in Figure 4A, cells showed cytotoxic response to scopolamine treatment, and when cultured in the presence of i-Extract, rich in withaferin A and withanone (Figure 4B) supplemented medium exhibited recovery (Figure 4A). As shown in Figure 4C, scopolamine also caused cell death in neuronal (IMR32) cells. Cytotoxicity as seen by appearance of apoptotic cells was confirmed by TUNEL assay. Cells pre-treated and recovered in medium supplemented either with i-Extract or its component withanone showed recovery from apoptosis (Figure 4D). Withaferin A did not protect the cells against scopolamine toxicity (Figure 4C and 4D; and data not shown).

### Glial cell protection by i-Extract and withanone

**Figure 5:**
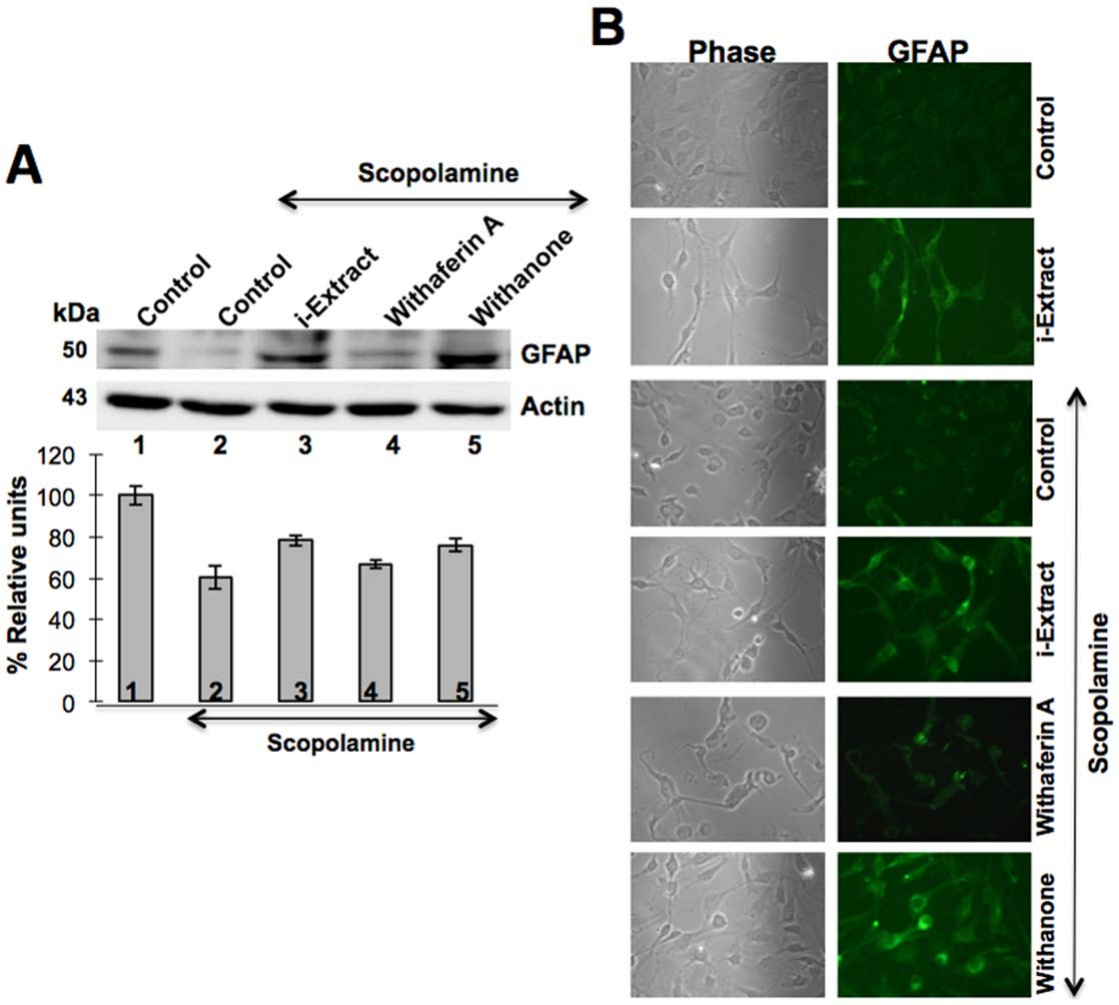
Ashwagandha i-Extract and withanone treatment and recovery of glial cell differentiation marker GFAP in scopolamine-treated C6 cells. Level of GFAP expression in C6 glioma cells treated with either scopolamine or scopolamine and i-Extract/withaferin A/withanone as described in material and methods. Levels detected by western blotting (A) and immunocytostaining (B) are shown. Quantitation from two independent experiments was performed using SigmaStat (Aspire Software International). Actin was used as an internal control.

In order to investigate whether the presence of i-Extract and withanone lead to recovery from scopolamine-induced toxicity associated with neuro/glial cell differentiation, we examined the levels of GFAP in i-Extract and scopolamine treated cells (Figure 5A). As shown, scopolamine treated cells showed decrease in GFAP expression. Of note, GFAP expression recovered when cells were pretreated either with i-Extract or withanone. Furthermore, i-Extract and withanone treated cells showed astrocytic extensions, characteristic of glial cell differentiation (Figure 5B). Withaferin A-treated cells did not show such astrocytic differentiation (Figure 5B).

### Neuronal cell protection by i-Extract and withanone

**Figure 6:**
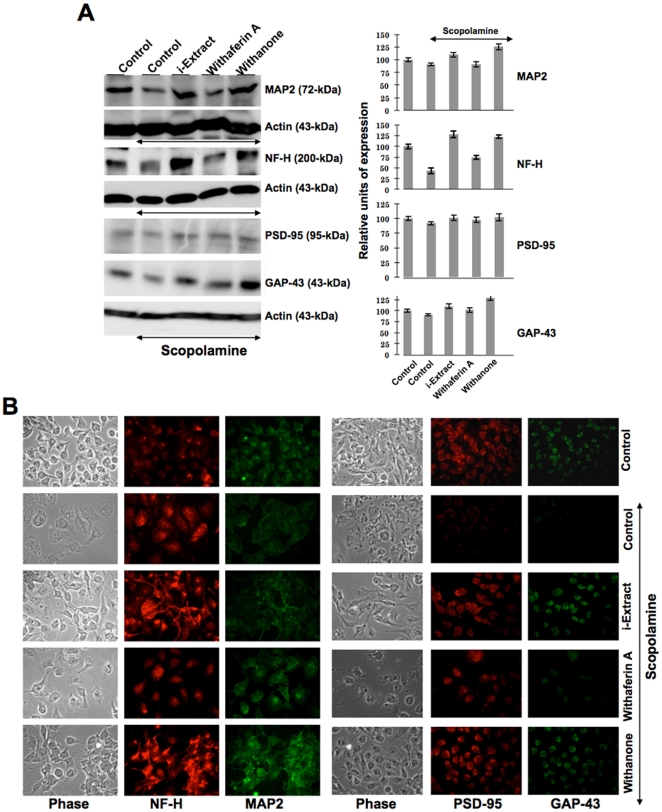
Recovery of neuronal cell differentiation markers in scopolamine-treated cells in response to Ashwagandha i-Extract and withanone treatment. Level of MAP2, NF-H, GAP43 and PSD-95 expression in IMR32 neuroblastoma cells treated with either scopolamine or scopolamine and i-Extract/withaferin A/withanone as described in material and methods. Levels of expression as detected by western blotting (A) and immunocytostaining (B) are shown. Quantitation was performed as described for Figure 5.

We examined the neuronal cell markers in IMR32 cells treated with scopolamine, i-Extract and withanone. As shown in Figure 6A and 6B, neuronal cell markers MAP2 (Microtubule-associated protein), NF-H (Neurofilament NF-H), PSD-95 (Postsynaptic marker protein) and GAP-43 (Growth-associated protein 43, an intrinsic determinant of neuronal development and plasticity) exhibited downregulation in the scopolamine treated cells, however the cells pretreated and recovered either in i-Extract or in withanone-supplemented medium showed recovery of all the four neuronal cell markers (Figure 6A and 6B). Withaferin A-treated cells did not show such recovery either in the Western blot or immunostaining assays (Figure 6A and 6B).

### Protection of scopolamine-induced DNA and oxidative stress by i-Extract and withanone

**Figure 7:**
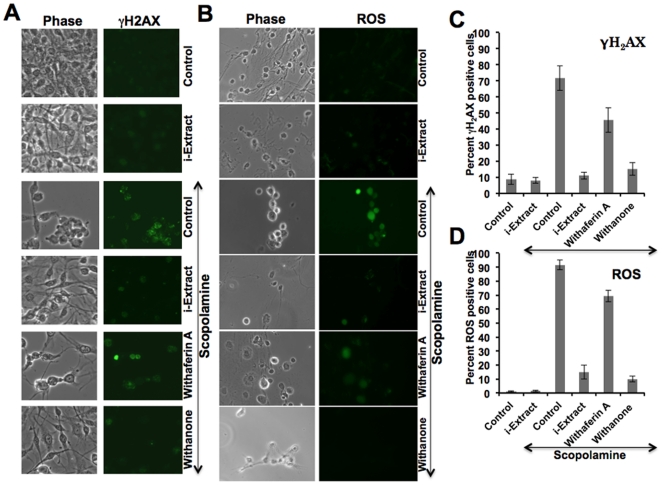
Treatment with Ashwagandha i-Extract/withanone and protection against scopolamine-induced DNA damage and oxidative stress. Level of expression of DNA damage marker (γH2AX) (A) and oxidative stress marker (reactive oxygen species, ROS) (B) in C6 cells treated with either scopolamine or scopolamine and i-Extract/withaferin A/withanone as detected by immunocytochemistry with specific antibodies and as described in material and methods are shown. Quantitation of γH2AX (C) and ROS (D) are shown, in which 200–300 cells were counted and scored with high intensity of signal.

We hypothesized that scopolamine may cause DNA and oxidative stress to cells and i-Extract may have protective function. In order to investigate the hypothesis, we examined DNA and oxidative damage induced proteins in cells treated with scopolamine and i-Extract. As shown in Figure 7A and 7B, scopolamine treated cells showed high levels of gamma γH2AX and ROS. Cells pre-treated and recovered in i-Extract supplemented medium showed decreased levels of these proteins and similar change was obtained with withanone (Figure 7C and 7D). On the other hand, withaferin A treated cells showed high levels of γH2AX, p53, p21 and ROS (Figure 7 and data not shown). These data suggested that i-Extract and withanone treated cells were protected against the DNA damage and oxidative stress caused by scopolamine.

## Discussion

Ashwagandha (*Withania somnifera:* solanaceae) is one of the widely used herbs in the Indian traditional system of medicine (Ayurveda). It is commonly referred to as ‘Indian Ginseng’. The extracts from different parts of the plant including roots, shoot and leaves have been claimed to have large variety of health promoting effects. The biologically active constituents of Ashwagandha leaves include alkaloids, steroidal lactones and saponins that have been proposed to possess anti-stress, anti-oxidant, analgesic, immunomodulatory, adaptogenic and immunostimulant properties. Some studies have also reported anticancer, neuroregeneration and cholinergic activities in Ashwagandha extracts [13]–[15]. Our present study showed that the scopolamine down-regulated neuroplasticity molecules and caused damage to glial and neuronal cells as assessed by the respective markers in mouse cerebrum and in C6 and IMR32 cell models. Furthermore, we found that the damage was dose and time dependent. These findings suggested cholinergic regulation of the neuroplasticity molecules as well as provide insight into the molecular basis of scopolamine-induced memory impairment. i-Extract attenuated scopolamine-induced down-regulation of neuronal and glial markers as well as recovered cells from oxidative stress. Such neuroprotective effect of i-Extract might be attributed to activation of muscarinic receptor mediated downstream signaling pathway and thereby transcriptionally regulating the neuroplasticity molecules.

### Scopolamine mediated down-regulation of BDNF and GFAP suggests alteration in their basal transcriptional machinery by M1 receptor signaling pathway

As demonstrated, scopolamine affected both neuronal and glial plasticity markers in dose and time dependent manner. It drastically reduced BDNF message (transcript variant 1) in mouse cerebrum. BDNF transcript variant 1 is neural tissue specific, expressed in all the regions of the brain [16] and associated with cognitive disorders like Alzheimer's disease [17]. Moreover, it is also reported that expression of transcripts containing exons I and III are most sensitive to activity-dependent regulation and are readily induced in response to synaptic activity [18]–[20]. Interestingly, the scopolamine-mediated down-regulation of BDNF message was transient and reverted back by 24 h to the level, close to that of control. Our results are consistent with earlier report on the involvement of cholinergic neuronal activity in the regulation of BDNF and NGF mRNAs in the developing and adult rat hippocampus [21]. Likewise, Kotani *et al* [22] observed that the scopolamine reduces BDNF message while cholinergic agonists (carbachol and pilocarpine) upregulate the mRNAs of BDNF and TrkB in the rat hippocampus. Some recent studies have shown that the cholinergic hypofunction induced by the toxin ^192^IgG-saporin impaired the memory acquisition, possibly through hippocampal Arc and BDNF down-regulation via muscarinic receptors [23]. Furthermore, in case of neurodegenerative disorders like Alzheimer's disease, Garzon *et al* [17] have observed 3–4 fold reductions in BDNF mRNA and protein levels in the hippocampus and cerebral cortex of human brain.

It is likely that the down-regulation of BDNF message by muscarinic antagonist scopolamine might be associated with M1 receptor mediated signaling pathway. Muscarinic acetylcholine receptors (mAChRs) belong to the G-protein-coupled receptor superfamily that transduces signals through PKC, Ca^2+^, cAMP and MAP kinase pathway [24]–[26], and thus modulate neuronal functions including long-term potentiation and synaptic plasticity implicated in learning and memory [27].

It has been established that the BDNF expression is controlled by the Ca^2+^ regulated transcription factor CREB that remains bound to BDNF promoter in an inactive form. Phosphorylation of CREB by calcium-regulated kinase cascades stimulates the recruitment of components of the basal transcription machinery to the BDNF promoter, and then new BDNF mRNA is synthesized [28]. On the other hand, drastic reduction in BDNF level by scopolamine could also be linked to scopolamine-mediated down-regulation of presenilins [3] that directly or indirectly through amyloid β influences transcriptional activity of CBP that in turn regulates the CREB-mediated basal transcriptional machinery of BDNF [29]. Since CREB signaling has been reported to undergo modulation by various factors related to different biological processes, there is a possibility that more complex mechanism is involved in the scopolamine-mediated alterations in BDNF message level. Our results also indicated that the scopolamine has transient effects on neuroplasticity molecules. French *et al* [30] also reported that the upregulation of the BDNF message by cholinergic agonists disappeared after 24 h. Such transient effects of scopolamine can be well correlated with its short-term impairment of learning acquisition and memory.

We report for the first time that the scopolamine affects glial plasticity marker GFAP in dose and time dependent manner. Although cholinergic regulation of GFAP has not been studied, some studies regarding suppression of GFAP by antidepressants proposed the involvement of transcription factors pCREB and NFkB regulating the transcription of GFAP [31]. It has been reported earlier that the muscarinic receptors lead to proliferation of astroglial cells via activation of NFkB that elevated intracellular calcium level and stimulated PKC signaling pathway [32]. Altogether, we suggest that the scopolamine-induced down-regulation of GFAP message might be attributed to muscarinic control of transcription factors and alteration in Ca^2+^, PKC and MAP kinase pathway. Furthermore, it can be speculated that the scopolamine mediated downregulation of both the plasticity markers could be linked via a common M1 receptor mediated alteration in calcium homeostasis that in turn modulates basal transcriptional machinery, particularly CREB regulated transcription of BDNF and GFAP.

### i-Extract attenuates scopolamine induced down-regulation of BDNF and GFAP

We, for the first time, have investigated the neuroprotective effect of i-Extract on scopolamine-mediated alterations and our findings indicate that this extract markedly attenuates the effects of the muscarinic antagonist. Such attenuation is more effective when the extract is administered prior to scopolamine treatment. Similar observations were made by Hung *et al* [33], when heat shock pretreatment was given to prevent scopolamine-induced amnesia. Besides attenuating muscarinic antagonism, the extract also up-regulated BDNF message and protein levels. Similarly, the herbal extract of wild ginseng also increases the expression of BDNF mRNA in the rat hippocampus [34]. In case of BDNF, we have analyzed transcript variant 1 and total protein level. We found that the data on the message and the protein expression levels were not corresponding completely. Such discrepancy between message and protein level may be attributed to multilevel regulation of its expression through different BDNF mRNA isoforms (I–IV) that are all translated into a single protein [35]. Also, the mature BDNF exhibits more remarkable change than its precursor form that may be assigned to alterations in the activity of proteases [36] that cleave the precursor form into mature form.

Similar to our observation, earlier studies have also demonstrated the enhancement of GFAP protein level in response to i-Extract treatment in C6 glioma cell line [37]. However, the mode of neurochemical action of i-Extract was not clearly understood. Based on differential effects of Ashwagandha root extract on acetylcholinesterase activity and its enhanced M1 receptor binding in basal forebrain nuclei [38], [39], it can be suggested that Ashwagandha exerts its neuroprotective action by agonizing muscarinic receptors. One of the active constituents of Ashwagandha extract, withanolide, has a steroidal structure [11]. Ashwagandha might stimulate signal cascades similar to steroids, particularly estradiol that has also been shown to improve spatial and working memory via cholinergic mechanisms [40], [41]. Like estradiol, i-Extract might activate three kinase pathways (CaMKII, Akt and ERK/MAPK) that contribute to CREB phosphorylation to different degrees. Widodo *et al* [42] have reported the anticancer activities in i-Extract that may involve modulation of JAK- STAT and MAP kinase signaling pathways. Taken together, we propose that the i-Extract may attenuate scopolamine mediated alterations of both BDNF and GFAP expressions by alteration of CREB signaling, either directly by elevating intracellular calcium level, by affecting Ca^2+^/CAMK or via modulation of ERK/MAPK/CREB signal transduction pathway that is known to be regulated by muscarinic receptors [26].

From *in vitro* models of glial and neuronal cells, we found that the scopolamine caused damage to cells that was considerably recovered by pretreatment with i-Extract and its component withanone. As seen in Figures 4 and 5, the damaged rounded cells in response to scopolamine treatment were replaced with healthy cells with extended neurites when treated with i-Extract and withanone. On the other hand, neither the cell survival nor the molecular markers of glial as well as neuronal cells showed any recovery when cells were treated with withaferin A. As seen in Figure 6, in neuroblastoma cells, increase in MAP-2 was observed when scopolamine-treated cells were pretreated and recovered either in i-Extract or withanone-supplemented medium. It indicated neurite and axon growth that were also accompanied by an increase in the expression of GAP-43 protein. The latter plays a key role in the growth of axons and dendrites by reorganization of the membrane cytoskeleton. GAP-43 has been established as an important component for regenerative response in the nervous system and is present at high level in neuronal growth cones during development and axonal regeneration. Cells treated with i-Extract and withanone indeed showed small multipolar processes endorsing the increase in dendrites as shown previously [43]. Similarly, analysis of NF-H expression showed its increase when cells were pretreated and recovered either in i-Extract or withanone supplemented medium. Immunostaining revealed long processes indicating axonal growth (Figure 6). The cells treated with withaferin A were unable to induce MAP2, although a moderate increase in NF-H was observed. The data suggested that withanone was most effective in restoration of neuronal network. Decrease in PSD-95, an established post-synaptic density marker in dendrites, in scopolamine treated cells indicated impaired synaptic density and its recovery in i-Extract and Withanone treated cells suggested recovery in the synaptic functions. Of note, withaferin A also caused recovery in PSD-95 expression, although the changes in MAP2, NF-H and GAP43 were more pronounced with i-Extract and withanone. We also found that the i-Extract and withanone protected the cells against DNA and oxidative stress caused by scopolamine (Figure 7). These data demonstrated that withanone is a predominant active component that protected the cells against scopolamine-induced damage. Further studies are warranted to reveal molecular details and pathways involved in neuroprotective effects of i-Extract and withanone that would promote their use for neuronal dysfunctions associated with age and other pathologies.

## Materials and Methods

### Animals

Male Swiss albino strain mice of 12 weeks (25–30 g) inbred and maintained in the animal house of Zoology Department, Banaras Hindu University, Varanasi, India, were used similar to other studies [44]–[46]. They were kept at 24±2°C on a 12-h light/dark cycle. Food and water were provided *ad libitum*. All the animal experiments were approved by the institutional animal ethical committee, Banaras Hindu University, Varanasi, India.

### Cell culture

Glioma C6 (rat) and neuronal IMR32 (human) cell lines were obtained from the Cell Resource Center for Biomedical Research, Tohoku University, Japan. The cells were maintained in Dulbecco's Modified Eagle's Medium DMEM (Invitrogen) supplemented with 10% fetal bovine serum in a humidified incubator (37°C and 5% CO_2_). Cells (40–50% confluency) were pretreated with Ashwagandha leaf extract (i-Extract, 0.8 µg/ml), withaferin A (0.2 µg/ml) and withanone (i-Factor, 5 µg/ml) for 24 h as described previously [43]. After 24 h, scopolamine (3 mM) was added to the cells for 1 to 2 h. Culture medium was replaced either with normal medium or medium supplemented with i-Extract for another 24 h following which the cells were harvested and processed for different assays.

### Drug treatment

To determine the dose dependent effects of scopolamine hydrobromide on memory function, increasing doses of scopolamine (1 mg, 3 mg, 6 mg and 10 mg per kg BW) dissolved in 0.9% normal saline (vehicle) was intraperitoneally administered to mice. In many other studies, intraperitoneal injection of scopolamine to rodents has been considered a reliable mode of drug administration [47]–[49]. After 3 h of drug administration, the cerebrum (cerebral cortex and underneath limbic structures) was dissected out.

To determine the time dependent effect, mice were intraperitoneally injected with scopolamine (3 mg/kg BW) and cerebrum was dissected after 1, 3, 6 and 24 h of treatment. Furthermore, to assess the effect of i-Extract on scopolamine induced changes, mice were divided into five groups. Group 1 mice were administered with normal saline, Group 2 mice with normal saline followed by scopolamine (3 mg/kg BW), and Group 3, 4 and 5 mice with increasing doses of i-Extract (100 mg, 200 mg, 300 mg/kg BW) orally followed by intraperitoneal injection of scopolamine (3 mg/kg BW) after 2 h. All the five groups of mice were treated for 7 days and then cerebrum was removed after the last administration. Furthermore, to assess the neuroprotective, therapeutic efficacy as well as per se effect of i-Extract, its optimum dose of 200 mg/kg BW was orally administered prior to, after and without scopolamine treatments, respectively, for 7 days.

### RNA isolation

Total RNA was isolated from the cerebrum of mice of different experimental groups using TRI Reagent (Sigma, USA) following the manufacturer's instruction. It was estimated by taking absorbance at 260 nm and RNA samples with A260/280≥1.8 were used further. Total RNA from different groups was resolved on 1.2% agarose formaldehyde gel and the integrity of RNA was checked by ethidium bromide staining of 18S and 28S rRNA.

### RT-PCR

RNA isolated from the cerebrum of mice of different experimental groups was first reverse transcribed into cDNA using reverse transcriptase. The resulting cDNA was used as templates for subsequent PCR amplification using specific primers for BDNF, GFAP and GAPDH as internal control [50], [51]. Primer sequence and PCR conditions are mentioned in Table 1. The PCR products were resolved on 2% agarose gel. The signals were scanned by AlphaImager system and analyzed by Alpha-EaseFC software (Alpha Innotech Corp, USA). After extraction of total RNA from mouse cerebrum, reverse transcription was performed. Five microgram of RNA from each experimental group was reverse transcribed and the resulting single-strand cDNA was amplified by PCR, which was performed with specifically designed primers for the genes in the study. For each gene (BDNF and GFAP) specific primers were used and amplified separately using optimal annealing temperature, number of cycles and annealing conditions, as mentioned in Table 1. Parallel to this reaction, same master mix of PCR reaction (cDNA, buffer, dNTP, H_2_O, Taq Polymerase) was used with a pair of glyceraldehyde 3-phosphate dehydrogenase (GAPDH)-specific primers to perform PCR of GAPDH with its specific annealing temperature, number of cycles and annealing conditions (mentioned in Table 1) as an internal control for normalizing variations in RNA aliquots taken for RT reactions and gel loading. PCR products were analyzed by electrophoresis on 2% agarose gel and stained with ethidium bromide. Band intensity was analyzed by densitometer.

**Table 1:** Gene specific primer sequences and corresponding PCR conditions.

Gene name/Amplicon length	Primer Sequences		Annealing conditions			Number of cycles
			Denature	Anneal	Extend	
**GAPDH/108 bp**	Forward	5′ GTCTCCTGCGACTTCAG-3′	94°C 30 s	52°C 30 s	72°C 30 s	26
	Reverse	5′-TCATTGTCATACCAGGAAATGAGC-3′				
**GFAP/641 bp**	Forward	5′-TTCCTGTACAGACTTCTCC-3′	94°C 60 s	52°C 30 s	72°C 45 s	29
	Reverse	5′-CCCTTCAGGACTGCCTTAGT-3′				
**BDNF/692 bp**	Forward	5′-TGCCAGAGCCCCAGGTGTGA-3′	94°C 60 s	63°C 30 s	72°C 45 s	32
	Reverse	5′-CTGCCCTGGGCCCATTCACG-3′				

### Immunostaining

Cells were cultured and treated on glass coverslips placed in 12-well culture dish. At the end of the treatment, coverslips were washed with cold phosphate-buffered saline (PBS) and the cells were fixed with pre-chilled methanol∶acetone (1∶1 v/v) mixture for 5–10 min. Fixed cells were washed with PBS, permeabilized with 0.2% Triton X-100 in PBS for 10 min, and blocked with 2% bovine serum albumin (BSA) in PBS for 20 min. Cells were stained with anti-GFAP (Sigma), anti-NF-H (Sigma), anti-MAP2 (Sigma), anti-PSD-95 (Santa Cruz), anti-GAP 43 (Santa Cruz), γH2AX (Millipore) antibodies. Immunostaining was visualized by secondary staining with Alexa-488 conjugated goat anti-rabbit antibody (Molecular probes). After three to four washings with 0.2% Triton X-100 in PBS (PBST), cells were overlaid with Fluoromount (Difco) and examined under Carl Zeiss microscope using epifluorescence optics. TUNEL staining was performed on cells grown and treated onto coverslips using the ApopTag® Red In Situ Apoptosis Detection kit (Millipore, Billerica, MA). ROS assay was performed as described previously [52]

### Western blotting

Mice were sacrificed by cervical dislocation and cerebrum was removed, homogenized in lysis buffer (50 mM Tris HCl pH 7.4, 1 mM EDTA, 120 mM NaCl and protease inhibitor cocktail) and centrifuged at 1000× g for 10 min. The supernatant was subjected to 15% SDS-PAGE, transferred to PVDF membrane, blocked in 5% skimmed milk/PBS, incubated overnight with primary antibody (anti-BDNF polyclonal rabbit antiserum 1∶1000, a gift from Dr Masami Kojima, AIST, Japan; anti-GFAP polyclonal rabbit antiserum 1∶1000 and anti-β tubulin isotype III 1∶5000 (Sigma), anti-GAPDH mouse monoclonal antiserum 1∶5000 (Santa Cruz Biotech), washed twice in 0.1% PBST, incubated in secondary antibody (goat-anti-rabbit IgG HRP 1∶2000 for BDNF and GFAP, and goat-anti-mouse IgG HRP 1∶3000 for tubulin and GAPDH), again washed twice in 0.1% PBST and finally subjected to ECL detection. The band intensities were measured by spot densitometry tool of AlphaEaseFC software (Alpha Innotech Corp, USA). For western blotting, cells were grown and treated in 6-well plates. Protein samples (10–20 µg, estimated by Bradford method) were harvested using Nonidet-P40 lysis buffer (20 mM Tris, 100 mM EDTA, 100 mM EGTA, 100 mM PMSF, 150 mM NaCl, and 1% NP-40), separated in SDS-polyacrylamide gels, and electroblotted onto PVDF membranes (Millipore) using a semidry transfer blotter (Biometra, Tokyo, Japan). Immunoblotting was performed with antibodies against GFAP (Sigma), actin (Chemicon International, Temecula, CA), NF-H (Sigma) and MAP-2 (Sigma). The immunoblots were incubated with horseradish peroxidase (HRP)-conjugated goat anti-mouse or anti-rabbit antibodies (Santa Cruz Biotech) and detected using ECL substrate (Amersham Pharmacia Biotech/GE Healthcare, Piscataway, NJ). Densitometric quantitation of the representative immunoblots was carried out using the ImageJ software (National Institute of Health).

### Statistical analysis

Each experiment was repeated three times (n = 9/group). For RT-PCR, the signal intensity of BDNF and GFAP message was analyzed after normalization against the signal intensity of internal control, GAPDH. The data are presented as a histogram with the mean (±SEM) of three values calculated as percentage relative density values (RDV) of BDNF/GAPDH and GFAP/GAPDH. For western blotting, the signal intensity of BDNF and GFAP was analyzed after normalization against the signal intensity of β-tubulin or GAPDH. The data are presented as a histogram with the mean (±SEM) of three values calculated as RDV of BDNF/β-tubulin, BDNF/GAPDH, GFAP/β-tubulin and GFAP/GAPDH. Statistical analysis was performed by one way analysis of variance (ANOVA) followed by post hoc tests of Student-Newman-Keuls method using SigmaPlot, version 2.0, Jandel Scientific Software. The data were reported as mean (± SEM) and P values<0.05 were considered as significant.
